# Triglyceride-glucose index as a novel prognostic biomarker for coronary artery disease: evidence from a large-scale prospective cohort study

**DOI:** 10.3389/fendo.2025.1653948

**Published:** 2025-09-11

**Authors:** Baoyu Feng, Yejing Zhao, Wei Xu, Xuyang Meng, Yi Li, Chenxi Xia, Zinan Zhao, Hongyu Peng, Xiang Wang

**Affiliations:** ^1^ Department of Clinical Trial Center, Beijing Tiantan Hospital, Capital Medical University, Beijing, China; ^2^ Department of Cardiology, Beijing Anzhen Hospital, Capital Medical University, Beijing, China; ^3^ Department of Geriatrics, Institute of Geriatric Medicine, Beijing Hospital, National Center of Gerontology, Chinese Academy of Medical Sciences, Beijing, China; ^4^ National Clinical Research Center for Cardiovascular Diseases, State Key Laboratory of Cardiovascular Disease, Fuwai Hospital, National Center for Cardiovascular Diseases, Chinese Academy of Medical Sciences and Peking Union Medical College, Beijing, China; ^5^ Department of Cardiology, Beijing Hospital, National Center of Gerontology, Institute of Geriatric Medicine, Chinese Academy of Medical Sciences, Beijing, China; ^6^ Department of Pharmacy, Beijing Hospital, National Center of Gerontology, Institute of Geriatric Medicine, Chinese Academy of Medical Sciences, Beijing Key Laboratory of Assessment of Clinical Drugs Risk and Individual Application (Beijing Hospital), Beijing, China

**Keywords:** triglyceride-glucose, coronary artery disease, acute coronary syndrome, chronic coronary syndrome, mortality, major adverse cardiovascular events

## Abstract

**Objective:**

The triglyceride-glucose (TyG) index, combining fasting glucose and triglyceride levels, has emerged as a promising biomarker for coronary artery disease (CAD). However, evidence for its long-term prognostic value in CAD remains limited and inconclusive.

**Methods:**

This prospective cohort study was conducted at Beijing Hospital between January 2016 and December 2021, involving 23,591 patients with CAD. Based on the inclusion and exclusion criteria, a total of 11,325 CAD patients were included in the final analysis. TyG index was determined using the formula ln [fasting triglycerides (mg/dL) × fasting glucose. Eligible participants were stratified by TyG tertiles: Tertile 1 (TyG ≤ 8.39, n=3,775); Tertile 2 (8.40 ≤ TyG ≤ 8.92, n=3,776); Tertile 3 (TyG ≥ 8.93, n=3,774), with a median follow-up duration of 28 months. The primary outcomes were all-cause death and cardiovascular disease (CVD) death. The secondary outcome was major adverse cardiovascular events (MACE). Cox proportional hazards models and restricted cubic spline (RCS) analysis were applied to investigate the relationship between the TyG index and endpoints.

**Results:**

RCS analyses showed a monotonic increase in all-cause death, CVD death, and MACE risks with higher TyG. Kaplan-Meier curves confirmed worse survival in higher TyG tertiles. Multivariable-adjusted analysis revealed continuous TyG index was an independent risk factor for all-cause death (HR = 1.22; 95% CI 1.02-1.45) and CVD death (HR = 1.61; 95% CI 1.17-2.22). Using the lowest TyG index tertile as the reference (T1), the highest tertile (T3) group exhibited a 1.34-fold risk of all-cause death (95% CI 1.04-1.72), a 1.99-fold risk of CVD death (95% CI 1.23-3.21), and a 1.17-fold higher risk of MACE (95% CI 1.00-1.37), respectively. Subgroup analyses showed the continuous TyG index was significantly associated with the risk of all-cause death in acute coronary syndrome (ACS) (HR = 1.33, 95% CI 1.01-1.74) and CVD death in chronic coronary syndrome (CCS) (HR = 1.79, 95% CI 1.16-2.78). With T1 as the reference, patients with CCS in the T3 group had a 2.15-fold higher risk of CVD death (95% CI 1.10-4.23).

**Conclusion:**

The TyG index correlates with increased all-cause death, CVD death, and MACE risks in CAD, with prognostic value in both ACS and CCS, particularly in CCS. These findings establish TyG as a robust CAD biomarker, warranting further clinical research.

## Introduction

Coronary artery disease (CAD), the leading cause of non-communicable disease deaths worldwide, accounts for approximately 17.9 million fatalities annually. Caused by coronary atherosclerosis, it is clinically categorized as stable chronic coronary syndromes (CCS) and acute coronary syndromes (ACS) (1, 2). Despite remarkable advancements in pharmacotherapy and revascularization procedures, patients with CAD continue to be susceptible to recurrent adverse cardiovascular events, underscoring the persistent burden of the disease. Therefore, early identification of high-risk CAD patients is crucial for improving risk stratification and optimizing personalized therapeutic strategies.

Insulin resistance (IR), a hallmark of metabolic syndrome, serves as a pivotal risk multiplier, increasing the susceptibility to cardiovascular disease and augmenting the risk of major adverse cardiovascular events (MACE) (3, 4). The triglyceride-glucose index (TyG), a composite marker of fasting plasma glucose and triglyceride (TG) levels, is widely recognized as a reliable and specific indicator of IR in clinical practice. Recently, the TyG index has emerged as a promising biomarker due to its significant correlations with CAD (5, 6). Previous cross-sectional studies have shown that higher TyG index was significantly correlated with a high risk of cardiovascular disease (7–9). However, to date, robust evidence validating the predictive value of the TyG index for the long-term prognosis of CAD remains limited and inconclusive. Moreover, the role of the TyG index in predicting long-term outcomes across the distinct clinical spectrums of ACS and CCS remains even less understood.

Against this backdrop, we conducted a prospective cohort study involving 23,591 adults with CAD, who were followed up for 28 months, to explore and validate the predictive power of the TyG index for mortality and MACE. Furthermore, subgroup analyses were performed to evaluate the prognostic value of the TyG index across distinct CAD subtypes, including ACS and CCS.

## Materials and methods

### Ethics statements

This prospective observational cohort study conformed to the Declaration of Helsinki and was approved by the Ethics Committee of Beijing Hospital (Ethics Number: 2021BJYYEC-167-01). Written informed consents were obtained from all participants.

### Study design and population

**Figure 1:**
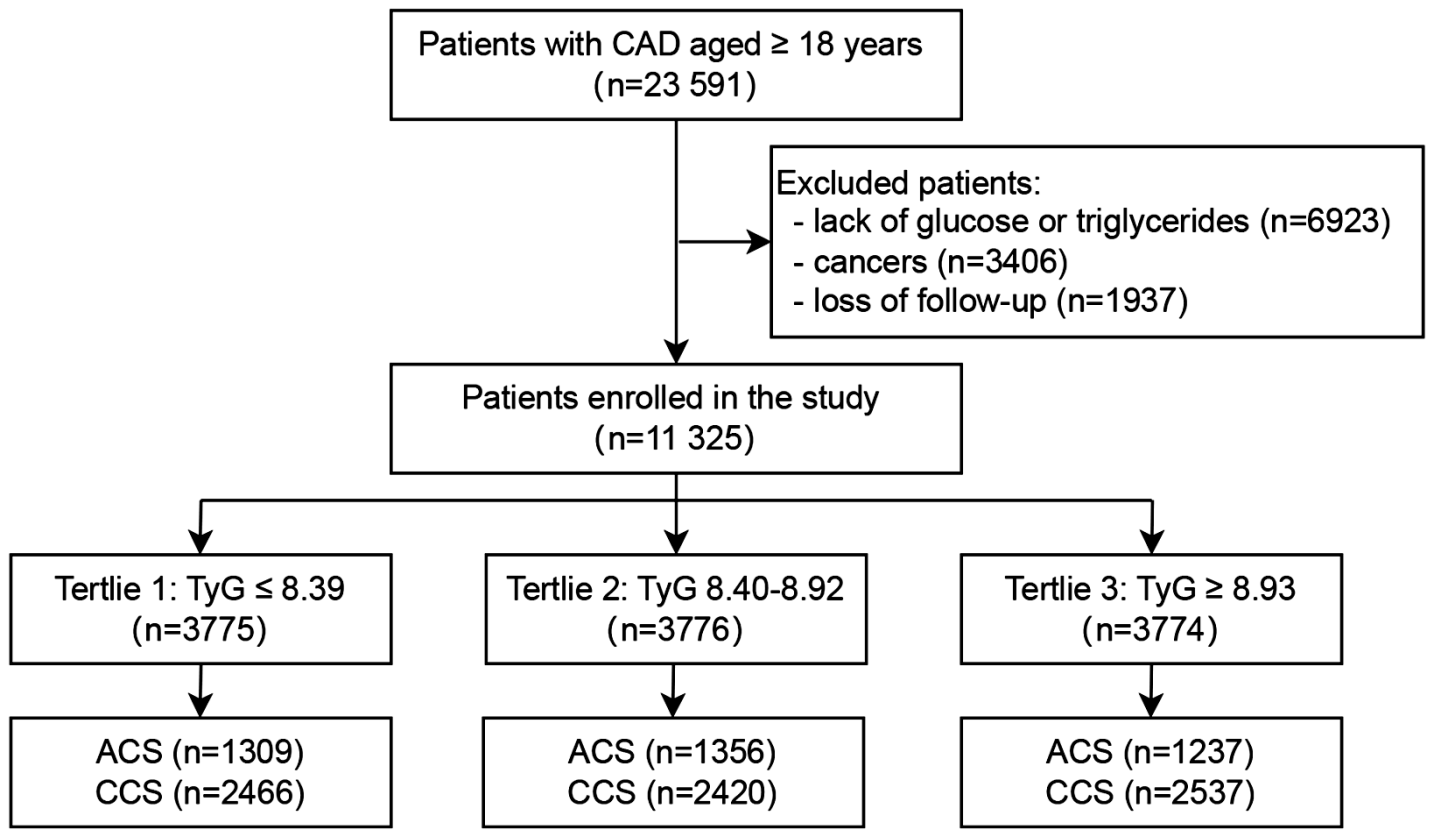
Flowchart of the study population. CAD, Coronary artery disease; TyG, Triglyceride-glucose; ACS, Acute coronary syndrome; CCS, Chronic coronary syndrome.

The study was conducted at Beijing Hospital in China between January 2016 and December 2021, involving 23,591 patients with confirmed CAD. Participants were excluded based on the following criteria: (1) Missing glucose or triglyceride data (n=6,923); (2) A diagnosis of cancer (n=3,406); (3) Loss to follow-up (n=1,937). A total of 11,325 patients were included in the final analysis (**Figure 1**). A comparison of baseline characteristics between excluded and included patients has been incorporated in the study, as detailed in **Supplementary Table S1**. According to the tertiles of the TyG index, these patients were divided into three groups: Tertile 1 (TyG ≤ 8.39, n=3,775); Tertile 2 (8.40 ≤ TyG ≤ 8.92, n=3,776); Tertile 3 (TyG ≥ 8.93, n=3,774).

### Data collection

General information of the subjects was collected, including age, gender, height, weight, systolic blood pressure (SBP), diastolic blood pressure (DBP), smoking and drinking history, past medical history (hypertension, diabetes, and hyperlipidemia), and the use of antidiabetic, antihypertensive, and hypolipidemic drugs. Body mass index (BMI) was calculated as weight in kilograms divided by height in meters squared. Morning fasting venous blood was collected, and related laboratory indicators were measured, including white blood cell (WBC), fasting blood glucose (FBG), total cholesterol (TC), TG, low-density lipoprotein cholesterol (LDL-C), high-density lipoprotein cholesterol (HDL-C), and glycohemoglobin (HbA1c). FBG, estimated glomerular filtration rate (eGFR), TC, TG, HDL-C, and LDL-C were measured using a LABOSPECT 008 system (Hitachi, Tokyo, Japan); the HbA1c level was determined via high-performance liquid chromatography (G8, TOSOH, Tokyo, Japan) at the laboratory of Beijing Hospital.

### Outcomes and follow-up

All participants were followed up with a median follow-up duration of 28 months (interquartile range [IQR]: 15–41 months). Clinical outcomes were collected and recorded during clinical follow-up visits. Outcome adjudication was independently conducted by two experienced clinicians uninvolved in baseline data collection, per a predefined standardized protocol. Adjudicators were blinded to participants’ baseline exposures, covariates, and study hypotheses, relying solely on outcome-related medical records. Discrepancies were resolved by a third senior cardiovascular expert, with their decision being final to minimize subjective bias. The primary outcomes were all-cause death and cardiovascular disease (CVD) death. The secondary outcome was MACE, defined as a composite outcome of CVD death, non-fatal myocardial infarction (MI), non-fatal stroke, heart failure hospitalization, and any revascularization procedure.

### Definitions

The TyG index was determined by the following formula: TyG = ln [fasting triglycerides (mg/dL) × fasting glucose (mg/dL)/2].

All-cause death was defined as the incidence of cardiac or non-cardiac death. CVD death was defined as fatal MI, fatal stroke, sudden death, and other cardiac death. MI was diagnosed in accordance with the contemporaneous Universal Definition of Myocardial Infarction (10). Any revascularization was defined as a revascularization of the target vessel or nontarget vessels.

### Statistical analysis

Continuous variables were summarized as means ± standard deviations (SD) or medians with interquartile ranges (IQR), and categorical variables were presented as counts and proportions. Baseline characteristics across TyG tertiles were compared using one-way analysis of variance (ANOVA) or the Kruskal-Wallis test for continuous variables, and the chi-square test for categorical variables. Restricted cubic spline (RCS) regression was employed to explore the relationship between TyG and all-cause death, CVD death, and MACE. Cox proportional hazards regression models were used to evaluate the association of TyG with all-cause death, CVD death, and MACE. The TyG index was analyzed as both a continuous variable and categorical tertiles (T1 ≤ 8.39, T2 8.40-8.92, T3 ≥ 8.93). Univariate and multivariable adjusted hazard ratios (HRs) were estimated using three models: Model 1 was unadjusted; Model 2 was adjusted for age and sex; and Model 3 was further adjusted for potential confounders, including BMI, SBP, smoking, drinking, ACS, TC, LDL-C, eGFR, and HR. Survival curves were plotted using the Kaplan-Meier curves, and differences among groups were compared by the log-rank test. Subgroup analyses were further performed to explore the association of TyG with outcomes stratified by CAD subtypes (ACS *vs*. CCS). All statistical analyses were conducted using SAS 9.4, and a two-tailed *P* value < 0.05 was considered statistically significant.

## Results

### Baseline characteristics of participants

A total of 11,325 patients with CAD were included in the final analysis (**Figure 1**). The average age of participants was 68 ± 11 years, and 63.7% were men. Patients were stratified into three groups based on TyG index tertiles: T1 (≤8.39, n=3,775), T2 (8.40–8.92, n=3,776), and T3 (≥8.93, n=3,774). Baseline characteristics were presented in **Table 1**. Compared with the other two groups, the highest tertile group (T3) was younger and exhibited higher levels of BMI, BP, HR, TC, TG, LDL-C, eGFR, and blood glucose. Additionally, patients in the T3 group had higher prevalences of hypertension, diabetes, dyslipidemia, and smoking, as well as higher utilization of hypoglycemic and antihypertensive medications (all *P*<0.05). The T2 group had a lower proportion of male patients. In terms of CAD subtypes, the T3 group had a lower proportion of ACS. Baseline characteristics among ACS and CCS population were presented in the **Supplementary Table S2**, **S3**.

**Table 1:** Baseline characteristics according to tertiles of the TyG index.

Variables	Total	T1(≤8.39)	T2(8.40-8.92)	T3(≥8.93)	*P* Value
N	11 325	3775	3776	3774	
Age, years	67.8 ± 11.1	70.1 ± 10.7	67.9 ± 10.7	65.3 ± 11.3	<0.001
Men, %	7217 (63.7)	2450 (64.9)	2346 (62.1)	2421 (64.1)	0.035
Smoking, %	4879 (43.1)	1567 (41.5)	1576 (41.7)	1736 (46.0)	<0.001
Drinking, %	6233 (55.0)	2049 (54.3)	2050 (54.3)	2134 (56.5)	0.074
BMI, kg/m^2^	25.6 ± 3.5	24.5 ± 3.4	25.8 ± 3.3	26.4 ± 3.5	<0.001
SBP, mmHg	136.2 ± 19.2	135.2 ± 19.1	136.4 ± 18.9	136.9 ± 19.5	0.002
DBP, mmHg	76.7 ± 12.0	75.7 ± 11.8	76.6 ± 11.7	77.7 ± 12.3	<0.001
HR, bpm	76.6 ± 13.4	76.0 ± 13.2	76.1 ± 13.4	77.7 ± 13.4	<0.001
TC, mmol/L	3.8 ± 1.0	3.5 ± 0.8	3.8 ± 0.9	4.1 ± 1.1	<0.001
TG, mmol/L	1.2 (0.9-1.7)	0.8 (0.6-0.9)	1.2 (1.1-1.4)	2.0 (1.6-2.5)	<0.001
LDL-C, mmol/L	2.2 ± 0.8	2.0 ± 0.7	2.2 ± 0.8	2.4 ± 0.9	<0.001
HDL-C, mmol/L	1.1 ± 0.3	1.2 ± 0.3	1.1 ± 0.2	1.0 ± 0.2	<0.001
HbA1c, %	6.8 ± 1.3	6.3 ± 0.9	6.6 ± 1.2	7.3 ± 1.5	<0.001
FBG, mmol/L	6.3 ± 2.1	5.2 ± 0.9	6.0 ± 1.4	7.7 ± 2.7	<0.001
eGFR, mL/min/1.73m^2^	87.8 (73.7-96.3)	87.1 (73.8-94.9)	87.7 (73.8-95.8)	88.9 (72.7-98.2)	<0.001
Hypertension, %	10083 (89.0)	3284 (87.0)	3378 (89.5)	3421 (90.6)	<0.001
Diabetes, %	5151 (45.5)	1170 (31.0)	1628 (43.1)	2353 (62.3)	<0.001
Dyslipidemia, %	10309 (91.0)	3275 (86.8)	3375 (89.4)	3659 (97.0)	<0.001
ACS, %	3902 (34.5)	1309 (34.7)	1356 (35.9)	1237 (32.8)	0.016
Antidiabetic drugs, %	3552 (31.4)	751 (19.9)	1095 (29.0)	1706 (45.2)	<0.001
Antihypertensive drugs, %	8155 (72.0)	2602 (68.9)	2754 (72.9)	2799 (74.2)	<0.001
Hypolipidemic drugs, %	9071 (80.1)	3000 (79.5)	3007 (79.6)	3064 (81.2)	0.119
Antiplatelet drugs, %	8559 (75.6)	2809 (74.4)	2851 (75.5)	2899 (76.8)	0.052
TyG	8.7 ± 0.6	8.0 ± 0.3	8.6 ± 0.2	9.4 ± 0.4	<0.001

Note: Data are means ± standard deviation, numbers (%), or medians (interquartile range).

Abbreviations: BMI, body mass index; SBP, systolic blood pressure; DBP, diastolic blood pressure; HR, heart rate; TG, triglyceride; TC, total cholesterol; LDL-C, low-density lipoprotein cholesterol; HDL-C, high-density lipoprotein cholesterol; HbA1c, glycosylated hemoglobin type A1C; FBG, fasting blood glucose; eGFR, estimated glomerular filtration rate; NGR, Normoglycemia; DM, diabetes mellitus; ACS, acute coronary syndrome; TyG, triglyceride glucose index.

The TyG index exhibited an approximate normal distribution (**Supplementary Figure S1**), with a mean ± SD of 8.7 ± 0.6. The mean ± SD values for the T1, T2, and T3 groups were 8.0 ± 0.3, 8.6 ± 0.2, and 9.4 ± 0.4, respectively.

### Association between the TyG index and clinical outcomes

**Figure 2:**
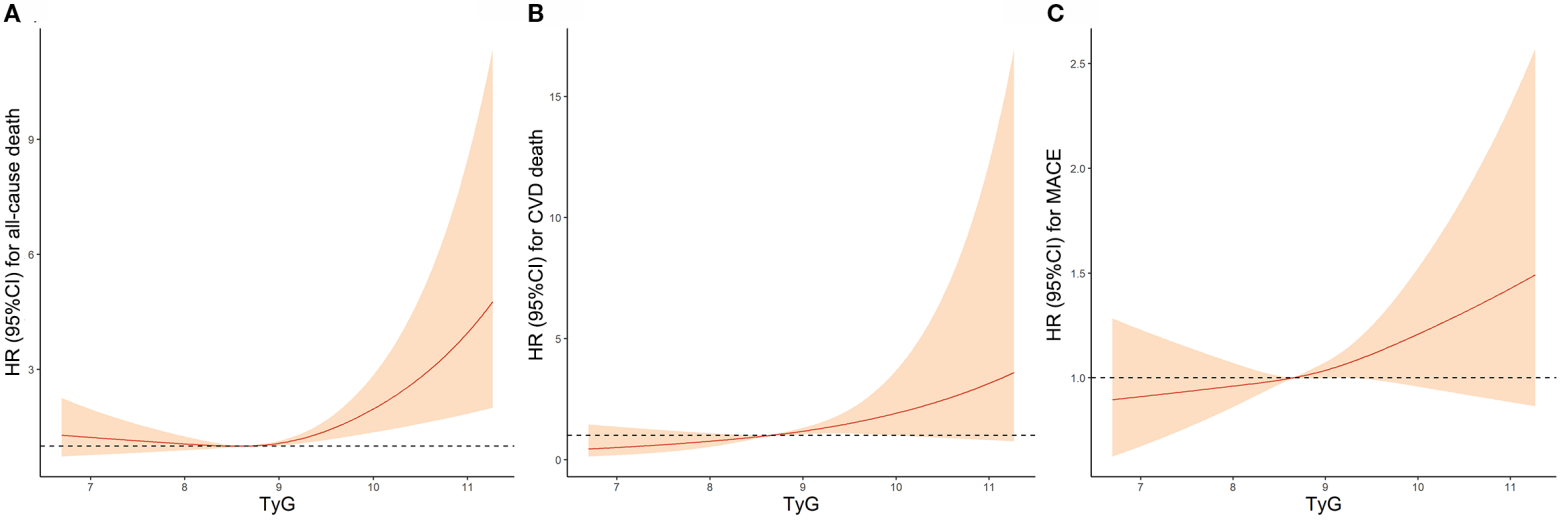
Association Between TyG Index and **(A)** all-cause death, **(B)** CVD death and **(C)** MACE by Restricted cubic spline analyses. TyG, Triglyceride-glucose; CVD, Cardiovascular disease; MACE, Major adverse cardiovascular events; HR, Hazard ratio; CI, Confidence interval.

Results from RCS analysis were presented in **Figure 2**. A monotonically increasing relationship was observed between the TyG index and risk of all-cause death (non-linear *P* = 0.007; **Figure 2A**), CVD death (non-linear *P* = 0.902; **Figure 2B**), and MACE (non-linear *P* = 0.570; **Figure 2C**).

**Figure 3:**
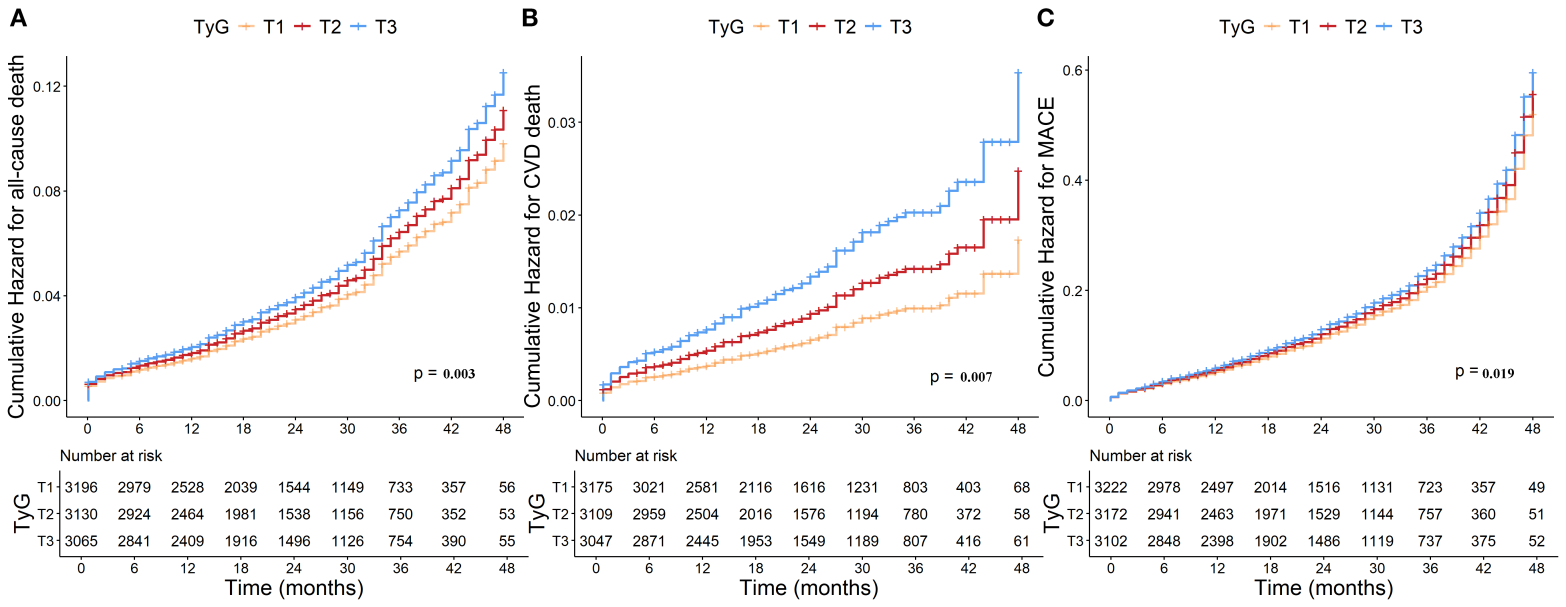
Kaplan-Meier curves for TyG index and **(A)** all-cause death, **(B)** CVD death and **(C)** MACE. TyG, Triglyceride-glucose; CVD, Cardiovascular disease; MACE, Major adverse cardiovascular events.

Numbers, events, and incidence density of all-cause death, CVD death, and MACE grouped by TyG tertiles were shown in the **Supplementary Table S4**. Kaplan-Meier survival analysis curves for all−cause death, CVD death and MACE stratified by TyG index tertiles were shown in **Figure 3**. In the overall population, significant differences in all-cause death (log-rank test *P* = 0.003; **Figure 3A**), CVD death (log-rank test *P* = 0.007; **Figure 3B**) and MACE (log-rank test *P* = 0.019; **Figure 3C**) were observed across the three groups.

The results of Cox regression analysis are presented in **Table 2**. In unadjusted model 1, the continuous TyG index was identified as an independent protective factor for all-cause death (HR = 0.86; 95% CI 0.78-0.96; *P* = 0.005), whereas no significant associations were observed with CVD death or MACE. Using the T1 group as the reference, the T2 group had a 24% lower risk of all-cause death (HR = 0.76; 95% CI 0.65-0.89; *P*<0.001) and a 12% lower risk of MACE (HR = 0.88; 95% CI 0.80-0.98; *P* = 0.022). The T3 group also exhibited a 17% lower risk of all-cause death (HR = 0.83; 95% CI 0.71-0.97; *P* = 0.013) compared with the T1 group. After further adjusting for potential confounders in model 3, the continuous TyG index emerged an independent risk factor for all-cause death (HR = 1.22; 95% CI 1.02-1.45; *P* = 0.029) and CVD death (HR = 1.61; 95% CI 1.17-2.22; *P* = 0.004). Additionally, when compared with the T1 group, the T3 group exhibited a 1.34-fold risk of all-cause death (95% CI 1.04-1.72; *P* = 0.024), a 1.99-fold risk of CVD death (95% CI 1.23-3.21; *P* = 0.005), and a 1.17-fold higher risk of MACE (95% CI 1.00-1.37; *P* = 0.047), respectively.

**Table 2:** Association of TyG with all-cause death, CVD death and MACE.

	Model 1		Model 3	
	HR (95%CI)	*P* value	HR (95%CI)	*P* value
All-cause death				
TyG	0.86(0.78-0.96)	0.005	1.22(1.02-1.45)	0.029
T1	Ref.		Ref.	
T2	0.76(0.65-0.89)	<0.001	0.83(0.65-1.07)	0.150
T3	0.83(0.71-0.97)	0.013	1.34(1.04-1.72)	0.024
CVD death				
TyG	1.09(0.89-1.33)	0.396	1.61(1.17-2.22)	0.004
T1	Ref.		Ref.	
T2	0.79(0.57-1.09)	0.151	1.07(0.65-1.76)	0.796
T3	1.19(0.89-1.60)	0.242	1.99(1.23-3.21)	0.005
MACE				
TyG	0.95(0.89-1.02)	0.150	1.11(1.00-1.23)	0.059
T1	Ref.		Ref.	
T2	0.88(0.80-0.98)	0.022	1.02(0.88-1.18)	0.829
T3	0.93(0.84-1.03)	0.165	1.17(1.00-1.37)	0.047

Model 1: univariate model; Model 2: adjust for age, and sex; Model 3: adjust for age, sex, BMI, SBP, smoking, drinking, ACS, TC, LDL-c, eGFR, and HR.

Abbreviations: TyG, triglyceride glucose index; CVD, cardiovascular disease; MACE, major adverse cardiovascular events.

### Subgroup analysis of ACS *vs* CCS

We performed subgroup analyses to investigate the consistency of the association between the TyG index and outcomes across ACS and CCS subgroups. The TyG index exhibited an approximate normal distribution in both ACS and CCS subgroups (**Supplementary Figure S1B, C**), with mean ± standard deviation (SD) values of 8.7 ± 0.6 and 8.7 ± 0.6, respectively.

**Figure 4:**
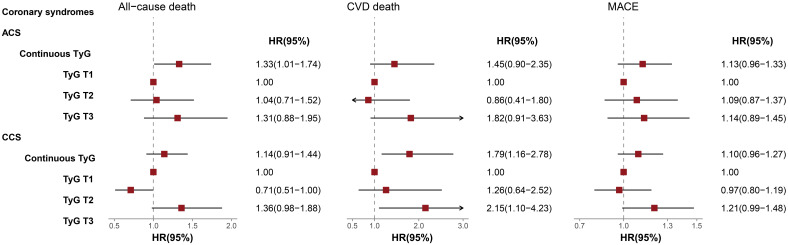
Multivariable-adjusted HRs of TyG index for Outcomes in ACS and CCS Subgroups. The interaction *P*-values for all-cause death, CVD death, and MACE were 0.208, 0.770, 0.438, respectively. TyG, Triglyceride-glucose; ACS, Acute coronary syndrome; CCS, Chronic coronary syndrome; CVD, Cardiovascular disease; MACE, Major adverse cardiovascular events; HR, Hazard ratio; CI, Confidence interval.

The results of subgroup analysis after further adjustment for potential confounders were presented in **Figure 4**. Within the ACS subgroup, the continuous TyG index was significantly associated with all-cause death risk (HR = 1.33, 95% CI 1.01-1.74). By contrast, among CCS patients, the continuous TyG index exhibited a significant association with CVD death risk (HR = 1.79, 95% CI 1.16-2.78). Moreover, patients with CCS in the T3 group had a 2.15-fold increased risk of CVD death compared with the T1 group (95% CI 1.10-4.23).

To verify the consistency and stability of the results, we also conducted subgroup analyses stratified by sex, renal function, and diabetes status, with the findings detailed in **Supplementary Figure S2**.

## Discussion

In this study, we conducted a 28-month prospective follow-up of 11,325 patients with CAD in China, to investigate the predictive value of the TyG index for long-term prognosis of CAD. The results revealed a significant association between the TyG index and risks of adverse clinical events in CAD patients, with a monotonically increasing relationship identified by RCS analysis. After adjusting for potential confounders, the TyG index was an independent predictor of all-cause death and CVD death. Compared with the lowest quartile group (T1) of the TyG index, the highest quartile (T3) was associated with significantly increased risks of all-cause mortality, CVD mortality, and MACE. Additionally, subgroup analysis demonstrated that the TyG index could predict mortality risk in both ACS and CCS populations, with a stronger association observed in CCS.

The state of IR triggers a cascade of metabolic disruptions contributing to the development of various chronic diseases including CAD. IR has been recognized not only as a pathogenic factor but also as a predictor of CAD in both general populations and individuals with diabetes (3, 11, 12). TyG, a biomarker derived from fasting glucose and triglyceride levels, has gained prominence as a surrogate for IR due to its straightforward calculation and high sensitivity/specificity (13). Consistent prior clinical data have shown that an elevated TyG index is associated with a higher incidence of CAD, highlighting its potential clinical utility in cardiovascular risk prediction (7–9). However, the existing evidence is fragmented, with most studies relying on cross-sectional designs. Current evidence regarding the predictive value of the TyG index for the long-term prognosis of CAD, particularly for mortality risk, remains inconclusive. We conducted this prospective cohort study involving a large sample of CAD patients with an average follow-up of 28 months, aiming to investigate the predictive value of the TyG index for long-term prognosis in CAD, with a particular focus on all-cause and CVD death risk. Our study suggested that a strong positive association between TyG index and risks of adverse clinical events in patients with CAD. The TyG index showed a protective effect in the unadjusted model but reversed to a risk factor in the adjusted models. This inversion was primarily due to the fact that the TyG index, as an “integrated marker of glucose and lipid metabolism”, is prone to fluctuations influenced by a combination of endogenous metabolic status, exogenous interventions, and baseline characteristics. The unadjusted model, which only captured the direct association between the TyG index and outcomes without controlling for confounders (e.g., age, gender, comorbidities, medication history), fails to account for potential interactions between these factors and the TyG index, thereby obscuring its true effect. Consequently, rigorous confounding control (e.g., multivariable adjustment, stratified analysis) is imperative in observational studies to accurately delineate its genuine association with disease outcomes. In our study, after adjusting for potential confounders, the continuous TyG index was an independent risk factor for all-cause death and CVD death. Specifically, each unit increase in the TyG index was associated with a 22% higher risk of all-cause mortality and a 61% higher risk of CVD death. Furthermore, when the TyG index was categorized into tertiles, the highest tertile (T3) was associated with a 34% increased risk of all-cause mortality, a nearly two-fold risk of CVD death, and a 17% increased risk of MACE compared with the lowest tertile (T1). These findings confirmed the predictive value of TyG index for the long-term prognosis of CAD, especially as a strong predictor of CVD death. However, several research findings remain inconsistent with our results, particularly the conflicting evidence concerning the predictive value for mortality risk. Yang et al. (14) included 5,489 non-diabetic patients who underwent percutaneous coronary intervention (PCI) and found that the TyG index was not an independent risk factor for adverse cardiovascular outcomes, including all-cause death, non-fatal MI, non-fatal stroke, and target vessel revascularization, after a median 28-month follow-up. Subgroup analysis revealed that in patients with LDL-C < 1.8 mmol/L, the TyG index was an independent hazard factor for the primary endpoint. The discrepancy in results may be attributed to differences in study populations and sample sizes. Yang et al.’s research included non-diabetic patients undergoing PCI with a relatively small sample size, whereas our study enrolled a larger cohort of CAD patients, nearly half of whom had diabetes. A meta-analysis of 12 studies performed by Luo et al. involving 28,795 CAD patients revealed that the highest TyG index quartile was associated with increased risks of MI, revascularization, and stroke compared with the lowest quartile. However, no significant differences were observed in all-cause mortality and CVD mortality risk analyses (15). Notably, most studies included in this meta-analysis focused on ACS with a small sample size, whereas our study primarily enrolled patients with CCS, a distinction that may underlie the divergent results. Meanwhile, another study by Jin et al. (16) involving the population with stable CAD, similar to our research cohort, reported a positive correlation between the TyG index and cardiovascular events (mortality and MI) over 36 months of follow-up, which is consistent with our research findings. Furthermore, we observed a non-linear association between the TyG index and the risk of all-cause death, characterized by “a relatively stable risk in the low TyG range and a significant increase in risk with higher TyG values in the high range”. This pattern may reflect the pathophysiological mechanisms of the “threshold effect” of insulin resistance and the “cumulative amplification” of metabolic disorders. In the low TyG range, the body maintains glucose and lipid homeostasis via compensatory mechanisms, with insulin resistance causing no significant vascular or organ damage, hence no marked elevation in mortality risk. Once the TyG index exceeds the critical threshold, insulin resistance enters a decompensated stage, exacerbating oxidative stress, inflammation, and metabolic remodeling. Concurrently, high TyG values often coexist with metabolic syndrome components (abdominal obesity, hypertension, diabetes), whose synergistic effects in the high range “amplify” risks, ultimately increasing all-cause mortality. It not only reflects the cumulative effect of metabolic disorders but also provides a certain threshold reference for clinical risk intervention. In summary, the findings of ours underscore the pivotal role of IR and glucose-lipid metabolic disorders in deteriorating the prognosis of CAD. Elevated TyG index, a surrogate marker for IR and metabolic derangements, may precipitate a cascade of pathological mechanisms, including inflammation, oxidative stress, reduced nitric oxide (NO) bioavailability, endothelial dysfunction, and platelet hyperactivity, thereby impairing coronary microcirculation and myocardial energy metabolism, exacerbating CAD progression, and increasing the vulnerability to cardiovascular events (17–19).

In addition, we further conducted subgroup analysis to investigate the association between TyG index and prognostic risk in different subtypes of CAD. The results demonstrated that the TyG index served as an independent prognostic factor in both ACS and CCS, but its associations with specific types of death diverged significantly between the two groups. In the ACS subgroup, each 1-unit increase in the TyG index was significantly associated with a 33% higher risk of all-cause death, while no statistical differences were observed for CVD death or MACE. By contrast, in the CCS population, a 1-unit increase in the TyG index was linked to a 79% higher risk of CVD death, and the highest quartile group (T3) exhibited a 2.15-fold elevated risk of CVD death compared with the lowest quartile group (T1). These findings suggested that a high TyG index may exhibit a stronger association with the long-term prognosis of CAD patients with a chronic course. The divergence in these results of subgroup analysis may be primarily attributed to the distinct pathophysiological mechanisms underlying CCS and ACS. CCS is characterized by chronic myocardial ischemia due to coronary artery stenosis, with a prolonged disease course during which metabolic abnormalities typically persist and gradually deteriorate (1). Long-term IR impairs the activation of the PI3K/Akt signaling pathway while activating the MAPK pathway, leading to reduced nitric oxide (NO) production in vascular endothelial cells and increased expression of adhesion molecules. This promotes monocyte adhesion and foam cell formation, thereby accelerating the chronic progression of atherosclerotic plaques. Meanwhile, IR-induced defects in the insulin signaling pathway in cardiomyocytes result in decreased myocardial glucose uptake, forcing the heart to rely on fatty acid oxidation for energy supply. This leads to disturbances in cardiac energy metabolism and left ventricular remodeling, which may further culminate in cardiac death caused by conditions such as heart failure and arrhythmias (17, 20, 21). In contrast, ACS is defined by acute thrombosis secondary to plaque rupture, accompanied by a severe systemic inflammatory response. IR elevates free fatty acid levels, activates the NLRP3 inflammasome, and promotes the release of IL-1β and TNF-α. This triggers the infiltration of inflammatory cells within plaques and the activation of matrix metalloproteinases (MMPs), directly contributing to the degradation and rupture of the plaque fibrous cap. Furthermore, in the context of IR, platelet hyperreactivity and coagulation system activation can exacerbate “thrombus expansion” following acute thrombosis, amplifying myocardial ischemic injury. Beyond worsening myocardial ischemic injury, these factors may trigger a “metabolic-inflammatory synergism”, leading to systemic inflammation, multi-organ dysfunction, infectious complications, or stress-induced metabolic derangements, thereby increasing the risk of all-cause mortality (19, 22–24). This implies that the differing correlations of the TyG index with various death outcomes in ACS and CCS may essentially reflect two distinct pathological frameworks, namely acute inflammatory stress and chronic metabolic injury. Nevertheless, this hypothesis requires further exploration and validation in future studies. The findings highlighted the need for subtype-specific metabolic management strategies in CAD. For ACS, international guidelines have currently recognized “metabolic abnormalities” as a risk-enhancing factor. In the future, the TyG index may be incorporated into ACS risk stratification algorithms as an effective supplement to traditional assessment tools. For ACS patients with significantly elevated TyG, prolonging the course of intensive antithrombotic therapy or early initiation of GLP-1 receptor agonists (which possess both anti-inflammatory and metabolic regulatory properties) may reduce the risk of short-term recurrent ischemia. For CCS, the TyG index may serve as a valuable predictor of long-term prognosis, with improving IR as a core objective of cardiac protection. Proactive interventions to address glucose-lipid metabolic disorders could potentially mitigate adverse outcomes such as CVD death. However, all these require further validation with more data in future studies.

### Clinical implications and future directions

The TyG index, a marker of insulin resistance, blood lipid, and glycemic levels, shows a significant correlation with the risk of death and MACE. Although not currently included in standard cardiovascular risk assessments, the TyG index has emerged as a promising predictor of CAD prognosis. Its calculation relies on routine hospital measurements of blood lipids and glucose, making it a cost-effective tool for early identification of CAD patients with poor prognosis, as well as for improving risk stratification and optimizing treatment management.

However, further research is needed to validate our findings in larger multicenter cohorts and establish its clinical utility and optimal cut-off values. In addition, the pathological role of the TyG index in different sub-types of CAD still warrants further research. The potential benefits of TyG index-targeted treatments in CAD patients also require more in-depth validation.

The advantages of this study included a large sample size of CAD population in China, prospective study design, complete and long-term follow-up, and comprehensive adjustment for potential cardiovascular risk factors. Additionally, we explored whether this association generalized to different CAD subtypes. However, several limitations should be noted. First, our study utilized only baseline TyG measurements, which prevented capturing the potential impact of its dynamic variations on outcomes - creating a risk of either underestimating or overestimating long-term effects. As an indicator of dynamic glucose and lipid metabolism, TyG levels vary with disease progression, therapeutic interventions (such as modifications to glucose-lowering or lipid-lowering medications), and lifestyle alterations. Consequently, subsequent investigations will employ serial TyG monitoring and time-dependent Cox models to rigorously quantify its association with adverse outcomes. Second, despite adjusting for major confounders, the observational design could not fully eliminate residual confounding. Third, although the sample size was substantial, participants were from a single center in China, which may to some extent restrict the generalizability of the findings to other ethnicities or different healthcare systems. Future plans include multi-center, cross-ethnic studies with diverse populations across systems, using stratified analyses to explore potential heterogeneity in TyG-outcome associations and provide evidence for broader application.

In summary, our study demonstrates that the TyG index is significantly associated with increased risks of all-cause death, CVD death, and MACE in patients with CAD, with a monotonically increasing trend. The TyG index exhibits predictive utility for mortality risk in both ACS and CCS, and the association is notably stronger in the CCS subgroup. These findings suggest that the TyG index may act as a powerful prognostic biomarker in CAD, and underscore the critical need for prospective investigations into its clinical relevance and potential therapeutic applications.

## Data Availability

The original contributions presented in the study are included in the article/**Supplementary Material**. Further inquiries can be directed to the corresponding authors.

## References

[1] Knuuti J Wijns W Saraste A Capodanno D Barbato E Funck-Brentano C. 2019 ESC Guidelines for the diagnosis and management of chronic coronary syndromes. Eur Heart J. (2020) 41:407–77. doi: 10.1093/eurheartj/ehz425, PMID: 31504439

[2] Collaborators GBDCoD. Global, regional, and national age-sex-specific mortality for 282 causes of death in 195 countries and territories, 1980-2017: a systematic analysis for the Global Burden of Disease Study 2017. Lancet. (2018) 392:1736–88. doi: 10.1016/S0140-6736(18)32203-7, PMID: 30496103 PMC6227606

[3] Adeva-Andany MM Martinez-Rodriguez J Gonzalez-Lucan M Fernandez-Fernandez C Castro-Quintela E. Insulin resistance is a cardiovascular risk factor in humans. Diabetes Metab Syndr. (2019) 13:1449–55. doi: 10.1016/j.dsx.2019.02.023, PMID: 31336505

[4] Wang C Li F Guo J Li C Xu D Wang B. Insulin resistance, blood glucose and inflammatory cytokine levels are risk factors for cardiovascular events in diabetic patients complicated with coronary heart disease. Exp Ther Med. (2018) 15:1515–9. doi: 10.3892/etm.2017.5584, PMID: 29434736 PMC5776627

[5] Tao LC Xu JN Wang TT Hua F Li JJ. Triglyceride-glucose index as a marker in cardiovascular diseases: landscape and limitations. Cardiovasc Diabetol. (2022) 21:68. doi: 10.1186/s12933-022-01511-x, PMID: 35524263 PMC9078015

[6] Ramdas Nayak VK Satheesh P Shenoy MT Kalra S. Triglyceride Glucose (TyG) Index: A surrogate biomarker of insulin resistance. J Pak Med Assoc. (2022) 72:986–8. doi: 10.47391/JPMA.22-63, PMID: 35713073

[7] da Silva A Caldas APS Hermsdorff HHM Bersch-Ferreira AC Torreglosa CR Weber B. Triglyceride-glucose index is associated with symptomatic coronary artery disease in patients in secondary care. Cardiovasc Diabetol. (2019) 18:89. doi: 10.1186/s12933-019-0893-2, PMID: 31296225 PMC6625050

[8] Wang X Xu W Song Q Zhao Z Meng X Xia C. Association between the triglyceride-glucose index and severity of coronary artery disease. Cardiovasc Diabetol. (2022) 21:168. doi: 10.1186/s12933-022-01606-5, PMID: 36050734 PMC9438180

[9] Yang L Peng Y Zhang Z. The predictive value of triglyceride-glucose index for assessing the severity and MACE of premature coronary artery disease. Cardiovasc J Afr. (2024) 34:1–6. doi: 10.5830/CVJA-2023-060, PMID: 38407306

[10] Thygesen K Alpert JS Jaffe AS Chaitman BR Bax JJ Morrow DA. Fourth universal definition of myocardial infarction (2018). Eur Heart J. (2019) 40:237–69. doi: 10.1093/eurheartj/ehy462, PMID: 30165617

[11] de Oliveira Correia ET Mechanick JI Dos Santos Barbetta LM Jorge AJL Mesquita ET. Cardiometabolic-based chronic disease: adiposity and dysglycemia drivers of heart failure. Heart Fail Rev. (2023) 28:47–61. doi: 10.1007/s10741-022-10233-x, PMID: 35368233

[12] Hill MA Yang Y Zhang L Sun Z Jia G Parrish AR. Insulin resistance, cardiovascular stiffening and cardiovascular disease. Metabolism. (2021) 119:154766. doi: 10.1016/j.metabol.2021.154766, PMID: 33766485

[13] Simental-Mendia LE Rodriguez-Moran M Guerrero-Romero F. The product of fasting glucose and triglycerides as surrogate for identifying insulin resistance in apparently healthy subjects. Metab Syndr Relat Disord. (2008) 6:299–304. doi: 10.1089/met.2008.0034, PMID: 19067533

[14] Yang J Tang YD Zheng Y Li C Zhou Q Gao J. The impact of the triglyceride-glucose index on poor prognosis in nonDiabetic patients undergoing percutaneous coronary intervention. Front Endocrinol (Lausanne). (2021) 12:710240. doi: 10.3389/fendo.2021.710240, PMID: 34489866 PMC8417234

[15] Luo JW Duan WH Yu YQ Song L Shi DZ. Prognostic significance of triglyceride-glucose index for adverse cardiovascular events in patients with coronary artery disease: A systematic review and meta-analysis. Front Cardiovasc Med. (2021) 8:774781. doi: 10.3389/fcvm.2021.774781, PMID: 34926622 PMC8674619

[16] Jin JL Cao YX Wu LG You XD Guo YL Wu NQ. Triglyceride glucose index for predicting cardiovascular outcomes in patients with coronary artery disease. J Thorac Dis. (2018) 10:6137–46. doi: 10.21037/jtd.2018.10.79, PMID: 30622785 PMC6297409

[17] Yang Q Vijayakumar A Kahn BB. Metabolites as regulators of insulin sensitivity and metabolism. Nat Rev Mol Cell Biol. (2018) 19:654–72. doi: 10.1038/s41580-018-0044-8, PMID: 30104701 PMC6380503

[18] Molina MN Ferder L Manucha W. Emerging role of nitric oxide and heat shock proteins in insulin resistance. Curr Hypertens Rep. (2016) 18:1. doi: 10.1007/s11906-015-0615-4, PMID: 26694820

[19] Gerrits AJ Koekman CA van Haeften TW Akkerman JW. Platelet tissue factor synthesis in type 2 diabetic patients is resistant to inhibition by insulin. Diabetes. (2010) 59:1487–95. doi: 10.2337/db09-1008, PMID: 20200314 PMC2874710

[20] Riehle C Abel ED. Insulin signaling and heart failure. Circ Res. (2016) 118:1151–69. doi: 10.1161/CIRCRESAHA.116.306206, PMID: 27034277 PMC4833475

[21] Tan J Li X Dou N. Insulin resistance triggers atherosclerosis: caveolin 1 cooperates with PKCzeta to block insulin signaling in vascular endothelial cells. Cardiovasc Drugs Ther. (2024) 38:885–93. doi: 10.1007/s10557-023-07477-6, PMID: 37289375 PMC11438709

[22] Bell DS. Inflammation, insulin resistance, infection, diabetes, and atherosclerosis. Endocr Pract. (2000) 6:272–6. doi: 10.4158/EP.6.3.272, PMID: 11421545

[23] Wolf D Ley K. Immunity and inflammation in atherosclerosis. Circ Res. (2019) 124:315–27. doi: 10.1161/CIRCRESAHA.118.313591, PMID: 30653442 PMC6342482

[24] Large GA. Contemporary management of acute coronary syndrome. Postgrad Med J. (2005) 81:217–22. doi: 10.1136/pgmj.2004.022590, PMID: 15811883 PMC1743242

