# Supplementary material for: Triglyceride-glucose index as a novel prognostic biomarker for coronary artery disease: evidence from a large-scale prospective cohort study
Source: Front Endocrinol (Lausanne). 2025 Sep 11;16:1653948. doi: 10.3389/fendo.2025.1653948 (PMC12460115; doi:10.3389/fendo.2025.1653948)
Supplement: Supplementary file 1 [file DataSheet1.docx]

**Supplementary Tables and Figures**

***Supplementary Tables***

**Supplementary Table S1.** **Baseline characteristics according to follow-up status.**

| **Variables** | **Total** | **Loss of follow-up** | **Follow-up** | ***P* Value** |
| --- | --- | --- | --- | --- |
| N | 13 262 | 1937 | 11 325 |  |
| Age, years | 67.7±11.1 | 67.1±11.2 | 67.8±11.1 | 0.023 |
| Men, % | 8465 (63.8) | 1248 (64.4) | 7217 (63.7) | 0.552 |
| Smoking, % | 5866 (44.2) | 987 (51.0) | 4879 (43.1) | <0.001 |
| Drinking, % | 7297 (55.0) | 1064 (54.9) | 6233 (55.0) | 0.930 |
| BMI, kg/m^2^ | 25.6±3.5 | 25.7±3.6 | 25.6±3.5 | 0.272 |
| SBP, mmHg | 136.2±19.2 | 137.1±19.6 | 136.2±19.2 | 0.132 |
| DBP, mmHg | 76.7±11.9 | 77.3±11.7 | 76.7±12.0 | 0.098 |
| HR, bpm | 76.6±13.5 | 77.2±15.0 | 76.6±13.4 | 0.993 |
| TC, mmol/L | 3.8±1.0 | 3.9±1.0 | 3.8±1.0 | 0.003 |
| TG, mmol/L | 1.2 (0.9–1.7) | 1.2 (0.9–1.7) | 1.2 (0.9–1.7) | 0.007 |
| LDL-C, mmol/L | 2.2±0.8 | 2.3±0.9 | 2.2±0.8 | <0.001 |
| HDL-C, mmol/L | 1.1±0.3 | 1.1±0.3 | 1.1±0.3 | 0.049 |
| HbA1c, % | 6.8±1.3 | 6.8±1.5 | 6.8±1.3 | 0.761 |
| FBG, mmol/L | 6.3±2.1 | 6.4±2.1 | 6.3±2.1 | 0.429 |
| eGFR, mL/min/1.73m^2^ | 87.9 (73.7–96.3) | 88.8 (74.3–96.7) | 87.8 (73.7–96.3) | 0.111 |
| Hypertension, % | 11760 (88.7) | 1677 (86.6) | 10083 (89.0) | 0.002 |
| Diabetes, % | 8400 (63.3) | 1030 (53.2) | 7370 (65.1) | <0.001 |
| Dyslipidemia, % | 12058 (90.9) | 1749 (90.3) | 10309 (91.0) | 0.300 |
| ACS, % | 4523 (34.1) | 621 (32.1) | 3902 (34.5) | 0.040 |
| Antidiabetic drugs, % | 4188 (31.6) | 636 (32.8) | 3552 (31.4) | 0.198 |
| Antihypertensive drugs, % | 9487 (71.5) | 1332 (68.8) | 8155 (72.0) | 0.004 |
| Hypolipidemic drugs, % | 10558 (79.6) | 1487 (76.8) | 9071 (80.1) | <0.001 |
| Antiplatelet drugs, % | 9946 (75.0) | 1387 (71.6) | 8559 (75.6) | <0.001 |
| TyG | 8.7±0.6 | 8.7±0.6 | 8.7±0.6 | 0.006 |

Note: Data are means ± standard deviation, numbers (%), or medians (interquartile range).

Abbreviations: BMI, body mass index; SBP, systolic blood pressure; DBP, diastolic blood pressure; HR, heart rate; TG, triglyceride; TC, total cholesterol; LDL-C, low-density lipoprotein cholesterol; HDL-C, high-density lipoprotein cholesterol; HbA1c, glycosylated hemoglobin type A1C; FBG, fasting blood glucose; eGFR, estimated glomerular filtration rate; NGR, Normoglycemia; DM, diabetes mellitus; ACS, acute coronary syndrome; TyG, triglyceride glucose index.

**Supplementary Table S2. Baseline characteristics of patients with ACS.**

| **Variables** | **Total** | **T1 (≤8.39)** | **T2 (8.40-8.92)** | **T3 (≥8.93)** | ***P* Value** |
| --- | --- | --- | --- | --- | --- |
| N | 3902 | 1309 | 1356 | 1237 |  |
| Age, years | 66.9±10.6 | 69.1±10.5 | 66.9±10.3 | 64.4±10.4 | <0.001 |
| Men, % | 2716 (69.6) | 911 (69.6) | 931 (68.7) | 874 (70.7) | 0.544 |
| Smoking, % | 1859 (47.6) | 587 (44.8) | 649 (47.9) | 623 (50.4) | 0.020 |
| Drinking, % | 2344 (60.1) | 773 (59.1) | 826 (60.9) | 745 (60.2) | 0.613 |
| BMI, kg/m^2^ | 25.6±3.4 | 24.6±3.3 | 25.9±3.2 | 26.5±3.5 | <0.001 |
| SBP, mmHg | 135.1±18.5 | 133.7±18.4 | 136.2±18.0 | 135.6±19.0 | 0.002 |
| DBP, mmHg | 76.4±11.9 | 75.0±11.6 | 76.6±11.6 | 77.6±12.4 | <0.001 |
| TC, mmol/L | 3.7±0.9 | 3.4±0.8 | 3.6±0.9 | 4.0±1.0 | <0.001 |
| TG, mmol/L | 1.2 (0.9–1.7) | 0.8 (0.6–0.9) | 1.2 (1.1–1.4) | 2.0 (1.6–2.6) | <0.001 |
| LDL-C, mmol/L | 2.2±0.8 | 2.0±0.7 | 2.2±0.8 | 2.4±0.9 | <0.001 |
| HDL-C, mmol/L | 1.0±0.3 | 1.1±0.3 | 1.0±0.2 | 0.9±0.2 | <0.001 |
| HbA1c, % | 6.8±1.3 | 6.3±0.9 | 6.7±1.2 | 7.4±1.5 | <0.001 |
| FBG, mmol/L | 6.3±2.0 | 5.2±0.8 | 6.0±1.3 | 7.6±2.6 | <0.001 |
| eGFR, mL/min/1.73m^2^ | 83.0±20.3 | 82.5±19.2 | 82.7±20.2 | 83.9±21.4 | 0.003 |
| Antidiabetic drugs, % | 1324 (33.9) | 283 (21.6) | 435 (32.1) | 606 (49.0) | <0.001 |
| Antihypertensive drugs, % | 3145 (80.6) | 1016 (77.6) | 1117 (82.4) | 1012 (81.8) | 0.003 |
| Hypolipidemic drugs, % | 3563 (91.3) | 1192 (91.1) | 1226 (90.4) | 1145 (92.6) | 0.141 |
| Antiplatelet drugs, % | 3504 (89.8) | 1162 (88.8) | 1209 (89.2) | 1133 (91.6) | 0.040 |
| Hypertension, % | 3580 (91.7) | 1179 (90.1) | 1258 (92.8) | 1143 (92.4) | 0.024 |
| Diabetes, % | 1842 (47.2) | 433 (33.1) | 610 (45.0) | 799 (64.6) | <0.001 |
| Dyslipidemia, % | 3796 (97.3) | 1250 (95.5) | 1317 (97.1) | 1229 (99.4) | <0.001 |
| HR, bpm | 76.5±12.5 | 76.1±12.7 | 76.0±12.1 | 77.7±12.6 | 0.003 |
| TyG | 8.7±0.6 | 8.0±0.3 | 8.6±0.2 | 9.4±0.4 | <0.001 |

Note: Data are means ± standard deviation, numbers (%), or medians (interquartile range).

Abbreviations: BMI, body mass index; SBP, systolic blood pressure; DBP, diastolic blood pressure; TG, triglyceride; TC, total cholesterol; LDL-C, low-density lipoprotein cholesterol; HDL-C, high-density lipoprotein cholesterol; HbA1c, glycosylated hemoglobin type A1C; FBG, fasting blood glucose; eGFR, estimated glomerular filtration rate; NGR, Normoglycemia; DM, diabetes mellitus; HR, heart rate; ACS, acute coronary syndrome; TyG, triglyceride glucose index.

**Supplementary Table S3. Baseline characteristics of patients with CCS**

| **Variables** | **Total** | **T1 (≤8.39)** | **T2 (8.40-8.92)** | **T3 (≥8.93)** | ***P* Value** |
| --- | --- | --- | --- | --- | --- |
| N | 7423 | 2466 | 2420 | 2537 |  |
| Age, years | 68.2±11.3 | 70.6±10.8 | 68.4±10.9 | 65.8±11.7 | <0.001 |
| Men, % | 4501 (60.6) | 1539 (62.4) | 1415 (58.5) | 1547 (61.0) | 0.017 |
| Smoking, % | 3020 (40.7) | 980 (39.7) | 927 (38.3) | 1113 (43.9) | <0.001 |
| Drinking, % | 3889 (52.4) | 1276 (51.7) | 1224 (50.6) | 1389 (54.7) | 0.010 |
| BMI, kg/m^2^ | 25.5±3.5 | 24.5±3.4 | 25.8±3.4 | 26.4±3.5 | <0.001 |
| SBP, mmHg | 136.7±19.5 | 136.1±19.4 | 136.5±19.4 | 137.5±19.7 | 0.046 |
| DBP, mmHg | 76.8±12.0 | 76.1±11.9 | 76.6±11.8 | 77.7±12.2 | <0.001 |
| TC, mmol/L | 3.9±1.0 | 3.6±0.9 | 3.8±0.9 | 4.2±1.1 | <0.001 |
| TG, mmol/L | 1.2 (0.9–1.7) | 0.8 (0.6–0.9) | 1.2 (1.1–1.4) | 2.0 (1.6–2.5) | <0.001 |
| LDL-C, mmol/L | 2.3±0.9 | 2.0±0.7 | 2.3±0.8 | 2.5±0.9 | <0.001 |
| HDL-C, mmol/L | 1.1±0.3 | 1.2±0.3 | 1.1±0.3 | 1.0±0.2 | <0.001 |
| HbA1c, % | 6.7±1.3 | 6.2±0.9 | 6.6±1.2 | 7.3±1.6 | <0.001 |
| FBG, mmol/L | 6.4±2.2 | 5.2±0.9 | 6.0±1.5 | 7.8±2.8 | <0.001 |
| eGFR, mL/min/1.73m^2^ | 82.4±21.0 | 81.9±19.9 | 82.4±20.4 | 82.8±22.7 | <0.001 |
| Antidiabetic drugs, % | 2228 (30.0) | 468 (19.0) | 660 (27.3) | 1100 (43.4) | <0.001 |
| Antihypertensive drugs, % | 5010 (67.5) | 1586 (64.3) | 1637 (67.6) | 1787 (70.4) | <0.001 |
| Hypolipidemic drugs, % | 5508 (74.2) | 1808 (73.3) | 1781 (73.6) | 1919 (75.6) | 0.121 |
| Antiplatelet drugs, % | 5055 (68.1) | 1647 (66.8) | 1642 (67.9) | 1766 (69.6) | 0.096 |
| Hypertension, % | 6503 (87.6) | 2105 (85.4) | 2120 (87.6) | 2278 (89.8) | <0.001 |
| Diabetes, % | 3309 (44.6) | 737 (29.9) | 1018 (42.1) | 1554 (61.3) | <0.001 |
| Dyslipidemia, % | 6513 (87.7) | 2025 (82.1) | 2058 (85.0) | 2430 (95.8) | <0.001 |
| HR, bpm | 76.6±13.9 | 75.9±13.6 | 76.2±14.2 | 77.7±13.9 | <0.001 |
| TyG | 8.7±0.6 | 8.0±0.3 | 8.6±0.2 | 9.4±0.4 | <0.001 |

Note: Data are means ± standard deviation, numbers (%), or medians (interquartile range).

Abbreviations: BMI, body mass index; SBP, systolic blood pressure; DBP, diastolic blood pressure; TG, triglyceride; TC, total cholesterol; LDL-C, low-density lipoprotein cholesterol; HDL-C, high-density lipoprotein cholesterol; HbA1c, glycosylated hemoglobin type A1C; FBG, fasting blood glucose; eGFR, estimated glomerular filtration rate; NGR, Normoglycemia; DM, diabetes mellitus; HR, heart rate; ACS, acute coronary syndrome; TyG, triglyceride glucose index.

**Supplementary Table S4. Numbers, events, and incidence density of all-cause death, death caused by CVD, and MACE grouped by TyG tertiles.**

|  | **N** | **Events** | **Incidence density per 1000 person-years** |
| --- | --- | --- | --- |
| **All** |  |  |  |
| **All-cause death** |  |  |  |
| T1 | 3775 | 369 | 3.4 |
| T2 | 3776 | 284 | 2.6 |
| T3 | 3774 | 315 | 2.8 |
| **Death caused by CVD** |  |  |  |
| T1 | 3775 | 81 | 0.7 |
| T2 | 3776 | 64 | 0.6 |
| T3 | 3774 | 98 | 0.8 |
| **MACE** |  |  |  |
| T1 | 3775 | 739 | 7.0 |
| T2 | 3776 | 671 | 6.2 |
| T3 | 3774 | 722 | 6.5 |
| **ACS** |  |  |  |
| **All-cause death** |  |  |  |
| T1 | 1309 | 106 | 2.8 |
| T2 | 1356 | 97 | 2.5 |
| T3 | 1237 | 84 | 2.3 |
| **Death caused by CVD** |  |  |  |
| T1 | 1309 | 28 | 0.7 |
| T2 | 1356 | 23 | 0.6 |
| T3 | 1237 | 36 | 1.0 |
| **MACE** |  |  |  |
| T1 | 1309 | 231 | 6.3 |
| T2 | 1356 | 245 | 6.3 |
| T3 | 1237 | 234 | 6.4 |
| **CCS** |  |  |  |
| **All-cause death** |  |  |  |
| T1 | 2466 | 263 | 3.7 |
| T2 | 2420 | 187 | 2.6 |
| T3 | 2537 | 231 | 3.0 |
| **Death caused by CVD** |  |  |  |
| T1 | 2466 | 53 | 0.7 |
| T2 | 2420 | 41 | 0.6 |
| T3 | 2537 | 62 | 0.8 |
| **MACE** |  |  |  |
| T1 | 2466 | 508 | 7.3 |
| T2 | 2420 | 426 | 6.1 |
| T3 | 2537 | 488 | 6.6 |

Abbreviations: CVD, cardiovascular disease; MACE, major adverse cardiovascular events

***Supplementary Figures***


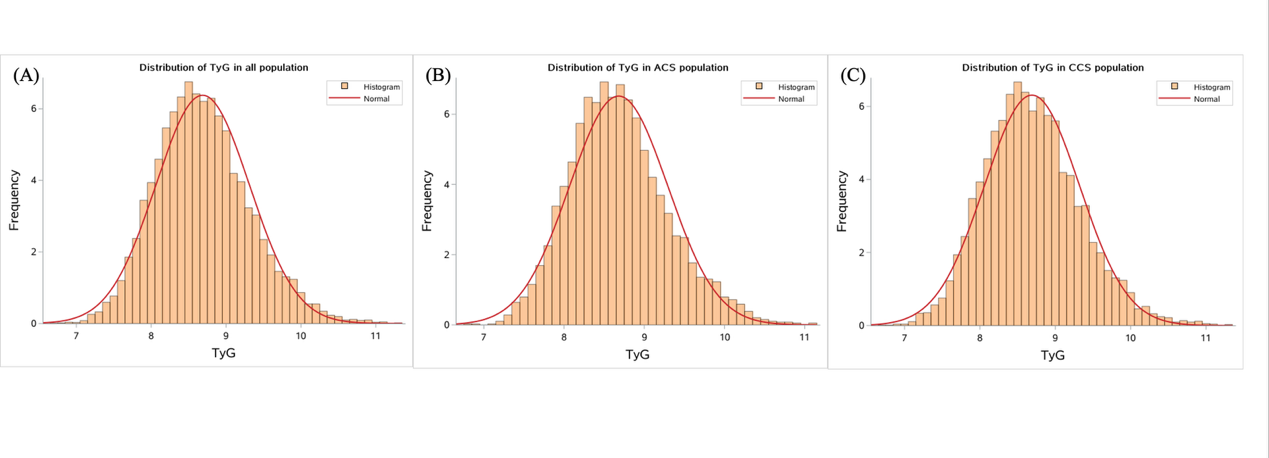
 Supplementary Figure S1. Distribution of the TyG index. (A) all population; (B) population with ACS; (C) population with CCS. TyG: Triglyceride-glucose, ACS: Acute coronary syndrome, CCS: Chronic coronary syndrome.


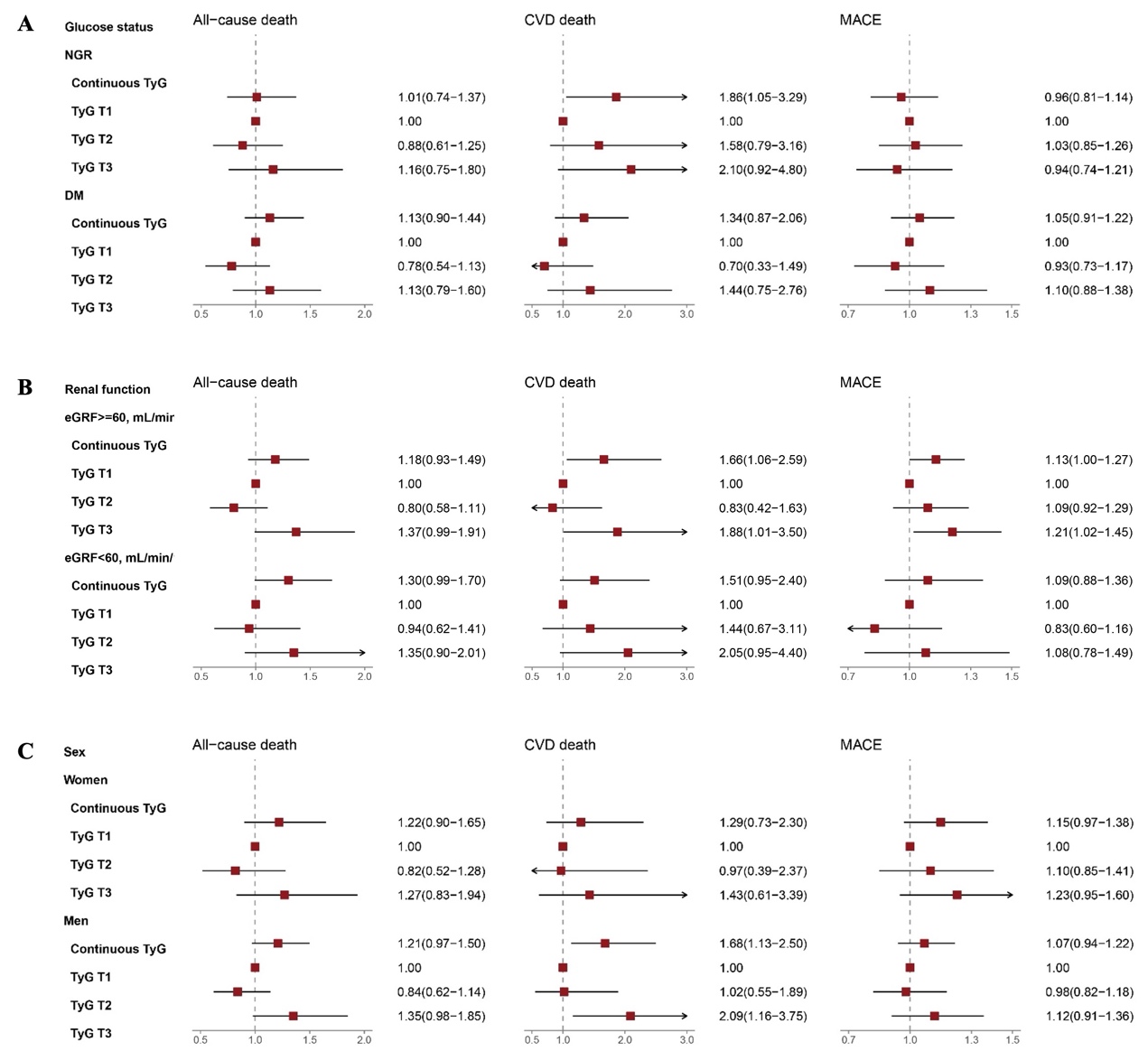


Supplementary Figure S2. (A) Subgroup analyses stratified by glucose status. The interaction *P*-values for all-cause death, CVD death, and MACE were 0.865, 0.421, 0.242, respectively. (B) Subgroup analyses stratified by renal function. The interaction *P*-values for all-cause death, CVD death, and MACE were 0.339, 0.464, 0.402, respectively. (C) Subgroup analyses stratified by sex. The interaction P-values for all-cause death, CVD death, and MACE were 0.996, 0.613, 0.660, respectively. TyG: Triglyceride-glucose, CVD: Cardiovascular disease, MACE: Major adverse cardiovascular events, NGR: Normal Glucose Regulation, DM: Diabetes Mellitus.
